# A Systematic Government-Driven Green Development Transformation Strategy with Chinese Characteristics: The Case Study of the Xining Metropolitan Area

**DOI:** 10.3390/ijerph20021321

**Published:** 2023-01-11

**Authors:** Jing Xu, Yongchun Yang, Zhuo Jia, Genying Chang, Yongjiao Zhang, Maoyuan Zhao, Wenrui Wang

**Affiliations:** 1 College of Earth and Environmental Sciences, Lanzhou University, Lanzhou 730000, China; 2 Key Laboratory of Western China’s Environmental Systems, Ministry of Education of the People’s Republic of China, Lanzhou University, Lanzhou 730000, China; 3 School of Economics, Lanzhou University, Lanzhou 730000, China

**Keywords:** systematic-driven, green development transformation, enforce, characteristic industries and industrial upgrading, Chinese characteristics, Xining metropolitan area (XMA)

## Abstract

In the 21st century, the tension between economic growth, resources and the environment in countries around the world is increasing, and the sustainable development of the economy and society is under great pressure. Green development has become the only way for countries to promote sustainable development. Generally, capitalist countries achieve their green development goals through increasingly strict environmental protection regulations, technological upgrading, industrial upgrading and global transfer based on market mechanisms and legal environments. Evidently, this green development strategy relies on the core position of Western countries in the global technological leadership and the global division of labor. However, limited in terms of their economic strength and by technical barriers, how can developing countries, led by China, in the marginal position in the global market competition, carry out green development transformation? In line with the “high-quality development” strategy, governments at all levels in China are actively exploring green development strategies with their own characteristics. Based on the Second Tibetan Plateau Scientific Expedition and Research and the face-to-face interview method, this paper summarizes a new strategy of systematic government-driven green development combining internal and external factors in the underdeveloped areas of inland China, which has gradually formed in the Xining metropolitan area (XMA) in the past 20 years. This strategy has the following characteristics: Firstly, during the period of rapid growth, the XMA areas have promoted each other through new urbanization and new industrialization and jointly promoted the formation of a green development turn in the new era. Secondly, the government is the core actor and driving force of China’s regional green development and has gradually formulated and implemented a series of policy systems during this development. Restricted by local economic backwardness and low industrial profits, the implementation of green government policies tends to be mandatory. The majority of urban residents and rural people support this transformation because they have benefited from the transformation process. Thirdly, this green development strategy is reflected in many aspects, such as industry, ecology, the environment, space and transportation, and is part of a systematic, green-oriented transformation. Fourthly, the advantages of the socialist system with Chinese characteristics are the guarantee of the green development strategy. It is noteworthy that this kind of green development transformation requires a large amount of “additional” investment and the “rapid” upgrade of the industry. Therefore, it requires more time and the understanding and assistance of all sectors of society.

## 1. Introduction

Since the 1960s, marked by ae “Silent spring” and “Limits to Growth”, countries around the world have gradually started to pay attention to the concept of sustainable development, conduct in-depth research on the concept of green development in combination with actual development and carry out effective research on energy efficiency and environmental pollution in the development of a green economy [1]. International organizations and scholars have actively explored national/local green development transformation strategies and green industrial policies in order to intervene in economic growth patterns and achieve coordinated economic, social and environmental development [2,3,4,5,6], such as the proposal of new concepts including “green finance”, “green building”, “green industry” and “green space” and active performance of the planning, design and construction of Green Cities, including ecological cities, livable cities, smart cities, resilience cities and sponge cities [7,8,9]. Pearce proposed the concept of the “green economy” in the book *Blueprint for a Green Economy*, advocated for an “affordable economy” [10], proposed including the costs of activities that are harmful to the environment and deplete resources in the national economic balance sheet and argued that economic development should fully consider the bearing capacity of the natural ecological environment. However, on a global scale, since the 1980s, based on neoliberalism, technological progress and increasingly stringent environmental protection standards, developed countries such as Europe and the United States have departed from a green development strategy based on the global market, technology network and trade system, relying on their technological leadership in the global market and their core position in the international division of labor [7,8,9]. First, they cleverly “transferred” their own ecological and environmental crisis through the international division of labor. They occupied both ends of the industrial “Smiling Curve” for a long time, relying on the rapid progression of emerging technologies, high-tech industries (clusters), technological protection measures and monopolistic competition. They not only obtained high profits through industrial upgrading/hollowing out but also cheaply consumed global natural gas and other clean energy/medium- and low-end products and “virtually” consumed the ecological and environmental resources (including carbon emissions) of developing countries. Secondly, they “forced” their companies/industries to improve the “greenness” of products/services by continuously improving their own environmental protection standards (such as resource utilization and the emission indices of pollutants) based on the strict legal environment. Thirdly, they tried to “constrain” the economic growth of developing countries through environmental issues such as carbon emissions, including the “Long Arm Jurisdiction” of the United States and the West, setting up environmental protection barriers and carbon emission thresholds. However, restricted in terms of their economic strength and by technical barriers, the vast number of developing countries mostly provide cheap raw materials and primary products to developed countries in the global market, whose production efficiency is low and results in great consumption and negative impacts on the ecological environment. Coupled with the dual pressures of rapid domestic population growth and economic growth, the green development and transformation of developing countries are under difficult to achieve.

Since its Reform and Opening Up, China’s rapid economic growth and its role as a “world factory” have not only consumed a great deal of domestic resources but also caused relatively serious environmental pollution and the degradation of water and soil environments, which profoundly reflects the logic of “development first, governance later”. This has attracted extensive attention in academic circles. First of all, Liu took the lead in proposing the concept of a “green economy” in China [11], which is the sustainable development economy with the coordinated development of the ecological economy as the core. Subsequently, scholars carried out research on green development strategies and policies, concepts and connotations, road and strategy transformation, strategic countermeasures and driving/restricting factors, measurement and evaluation index systems, theories and application methods [12,13,14,15,16,17,18]. Finally, scholars have discussed the green development transformation strategy from the perspectives of the influence mechanism, development path and enlightenment. Scholars have studied the impact mechanism by analyzing the relationship between institutions [19], markets [20], planning [20], technology [21] and green transformation development. Scholars have found that China’s green transformation development can be promoted through green economy policy institutions, green technology R&D and innovation, green industry energy structures and other paths [22], emphasizing the importance of government will, financial strength and the market [23].

Since 1978, Western China (including the Xining metropolitan area, or XMA) is the gathering/processing site of China’s minerals, energy and other bulk resources. Therefore, the regional development follows a trend of fast economic growth according to the urban growth coalition path dependency [24]. The damage/pollution of the ecological environment is particularly serious [25,26,27]. However, the attention and the effective governance of all sectors of life were rare before 2000 [28]. In fact, this is also the inevitable result of the fact that since the Third Plenary Session of the 11th Central Committee of the Communist Party of China proposed that the country should focus on “economic development”, local governments have only focused on rapid economic growth and ignored the constraints of the ecological environment. Since the beginning of the 21st century, the Chinese government has changed the previous development trend in terms of the thought and practice mode and put forward the concept of “ecological civilization”, advocated the construction of “Beautiful China”, actively implemented the requirements of the “Kyoto Protocol” and “carbon peaking and carbon neutrality goals” and actively promoted green development transformation. During this period, the central government has taken advantage of the socialist system with Chinese characteristics and adopted a “top-down” administrative “pressure” approach, for example, “sharing” the indicators to provinces and cities and “forcing” the implementation of green development. Local governments have also actively responded to the central government’s call to explore localized green development models [29]. Few studies have paid attention to the underdeveloped inland areas, especially the plateau towns with important ecological significance in terms of their location. However, the XMA is the only metropolitan area with a population of one million on the Qinghai–Tibet Plateau, and it is a typical nurturing metropolitan area. The social economy of the XMA has entered a new normal and is taking the road of green development. Taking it as an example and starting from the perspectives of the government, enterprises and residents, we explore a path of green development and transformation through the new Chinese urbanization of the plateau, which can help to provide reference for the less developed regions that are marginalized in the global market competition. The study also includes the following parts: first, the Materials and Methods; second, the systematic drive of the green development and transformation of the XMA; third, the actors and their basic characteristics regarding the green development and transformation of the XMA; fourth, the discussion; and, finally, the conclusion.

## 2. Materials and Methods

### 2.1. Research Area

**Figure 1:**
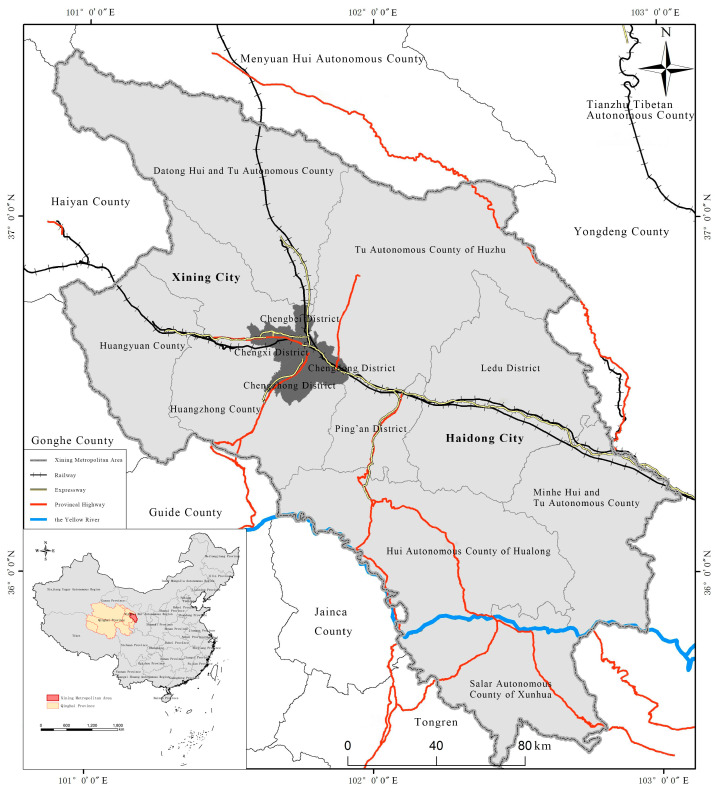
Location and Administrative Division of the XMA.

A metropolitan area is a geographical unit with one or more central cities as the core, relying on developed connection channels attracting surrounding cities or regions, with a highly integrated social economy [30,31]. By the end of 2021, China’s population urbanization level exceeded 64%, and its economic development and spatial organization have now fully entered the era of urban agglomerations/metropolitan circles leading local development. As an underdeveloped area in China’s inland areas, Western China urgently needs to build green, innovative and open metropolitan areas (such as the XMA) as regional growth poles. The XMA is located in Qinghai Province and roughly amounts to a commuting distance of 120 to 180 km, with a space range of vehicle traffic time from 1 to 1.5 h [32,33], encompassing seven districts and six counties in Xining and Haidong (Chengzhong District, Chengxi District, Chengdong District, Chengbei District, Huangzhong District, Datong Hui and Tu Autonomous County and Huangyuan County in Xining and Ledu District, Ping’an District, Minhe Hui and Tu Autonomous County, Tu Autonomous County of Huzhu, Salar Autonomous County of Xunhua and Hui Autonomous County of Hualong in Haidong) (Figure 1). In addition, here, we also clarify the location of the XMA in China, which further highlights the importance of the XMA.

At the end of 2020, the permanent resident population of the XMA was 3.8265 million (accounting for 64.95% of Qinghai Province). Xining’s population density was 186 people/km^2^ (much higher than the 8 people/km^2^ in Qinghai Province). Xining’s GDP was CNY 188.758 billion, and its average growth rate per annum was 3.9%. From 2000 to 2020, Xining’s RGDP was 9.20%, and the first degree of the population was 2.10, while the first degree of the GDP was 2.04. The ratio of the GDP of Qinghai Province increased from 57.05% to 62.8%, and the urban constructive land increased from 434.53 km^2^ to 549.19 km^2^, with an average annual increase of 1.22%. In the past 20 years, the XMA has developed significantly, and its internal structure and external form have taken shape rapidly. The typical characteristics of its development are its rapid economic growth, the emergence of industrial corridors, the market-oriented regional industrial division and agglomeration and the strengthening of regional connections. That is, its urbanization and industrialization processes are accelerating, its built-up areas are expanding rapidly, its ecological quality is improving, its living environment is improving, and its agglomeration of innovative elements is accelerating. This has improved the levels of livability and workability in the metropolitan area, as well as the creativity and socioeconomic ties inside and outside the metropolitan area, accelerated the industrial green transformation and factor agglomeration and improved the capacity for, and level of, green development [34,35]. Moreover, the XMA encompasses almost all the foreign-funded enterprises and globalization activities in Qinghai Province [36,37]. According to the “Lanzhou-Xining Urban Agglomeration Development Plan” issued by the State Council in 2018, the XMA is the only metropolitan area of new urban agglomeration with distinct regional characteristics and millions of people on the Qinghai–Tibet Plateau, and it is also one of the 11 cultivating metropolitan areas in China [38,39]. However, due to the limitation of the natural environment, the overall ecological environment of the metropolitan area is still relatively fragile, and ecological problems such as grassland degradation, land desertification and frequent natural disasters still exist. From 2016 to 2019, the green development level of XMA was higher than the overall level of Qinghai Province, and the level of Xining was higher than Haidong (except in 2019). On the county scale, the level of green development in the metropolitan area tends to be polarized, with a contrast between the high-value areas and low-value areas, which reflects the circular cumulative effect of the spatial distribution of green development in the XMA, and its green development is still at a relatively low stage of development [34]. As the “Chinese Water Tower”, the integral part of China’s ecological highlands and the important ecological function zone in the upper reaches of the Yellow River, the XMA has the uniqueness of typical plateau urbanization, and its green development transformation has inherent local restrictions. After 20 years of continuous exploration, a dual green development comprehensive system focusing on “high-quality growth” and ecological protection has been formed, which is an all-round, full-coverage and systematic green transformation strategy. Therefore, this study takes the XMA as a case in an attempt to summarize and analyze the green development strategy of underdeveloped inland areas in China and further promote the construction of an ecological civilization on the Qinghai–Tibet Plateau and provide experience of, and a reference for, green development in the similar regions of the world.

### 2.2. Research Methods

Relying on the Second Tibetan Plateau Scientific Expedition and Research, we used qualitative research methods and conducted three consecutive years of field trips and targeted face-to-face interviews in 2019–2021. The inspection methods mainly aimed to conduct government department interviews, enterprise interviews and field trips to visit major industries and facilities, including meetings with the leaders or supervisors of the development and reform commissions, the environmental protection administrations, the natural resource bureaus, the housing and urban–rural development bureaus, the science and technology bureaus, the industry and information technology bureaus, the investment invitation bureaus, the statistics departments, the open economic zones or industrial parks, the police bureaus and other relevant units of the cities and counties (including individual townships), as well as face-to-face interviews with supervisors from the development and reform commissions, the environmental protection administrations, the natural resource bureaus and some townships. The field trips included assessments of the green development and transformation of the districts and counties included in the XMA, mainly including the green development approaches and their existing problems, new urbanization, new industrialization, industrial upgrading and transformation, changes in the ecological environment and its governance, ecological migration, spatial governance and various related policies and plans.

For this research, we conducted 8 field trips. Among them, there were 2 large-scale field trips: one was a comprehensive field trip with a team of 12 people from August 6 to 18 in 2020, and the second was a comprehensive field trip with a team of 6 people from April 12 to 16 in 2021. During the investigation, 160 officials or supervisors participated in the seminars or interviews. Among them, Xining accounted for approximately 53%, and Haidong accounted for approximately 47%. We mainly visited the key local enterprises (such as those for brewing, dairy products, wolfberry processing and new energies and new materials), the industrial parks, the agricultural bases, the pollution control plants and key villages (such as villages and towns for ecological migration and rural revitalization). Additionally, we conducted panel discussions or open interviews with almost 110 entrepreneurs, managers, village leaders and villagers.

## 3. The Systematic Drive of the Green Development Transformation of the XMA

### 3.1. Green Industrialization and Green Urbanization: A Mutually Reinforcing Two-Way Cycle

**Figure 2:**
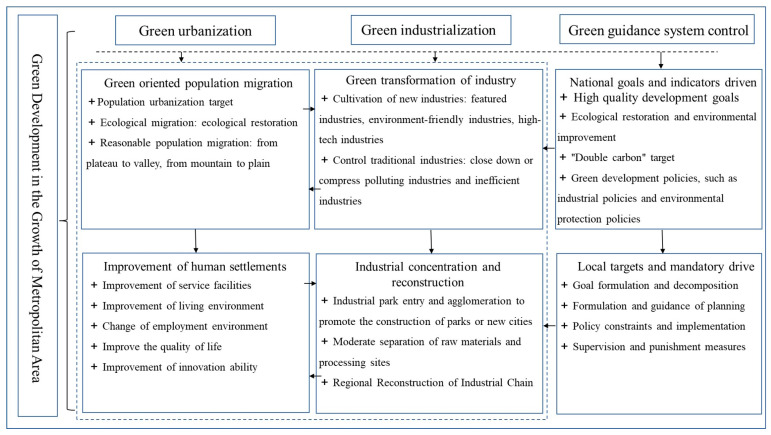
The Green Development Transformation System of the Mutually Reinforcing Two-way Cycle of the XMA.

The XMA has grown rapidly in the past 20 years, which is the result of the joint promotion of new urbanization and new industrialization: the “new” nature is the turn of green development in the new era. Firstly, this stems from the central government’s “sustainable development”/“high-quality development” strategy. Subsequently, through ecological restoration and environmental governance, “carbon peaking and carbon neutrality goals”, green industry policies and environmental protection policies, the State Council supervises the provinces, autonomous regions and cities in formulating green development plans, policies and measures by target decomposition, planning guidance, policy constraints, supervision and punishment measures. This is reflected especially in the exploration process of new industrialization and urbanization (Figure 2), and the two are always promoting each other [40].

Since 2000, based on the provincial strategic actions such as the national ecological barrier construction in Qinghai Province and “High-yield and High-benefit and High-quality” guidance, the “new industrialization” of the XMA has mainly been reflected in two aspects:

(1) Industrial upgrading: “the transformation of new and old kinetic energy”. On the one hand, the old kinetic energy should be compressed as much as possible, according to the locality’s “own timetable“. That is, the governments at all levels have adopted a series of policies, such as “close up”, “out of the city and into the park”, “increasing the threshold of industrial entry” and “technical upgrading/renovation”, to continuously eliminate large-scale, high-energy-consuming, high-pollution and low-efficiency enterprises and compress “black” industries and “close up” enterprises with a poor efficiency so that the proportion of traditional energy, non-ferrous metals and mining industries continues to slowly decline. From 2016 to 2019, the energy consumption per unit of GDP of Xining decreased by 30.26%. Thus far, Haidong has basically eliminated the titanium alloy industry and dismantled the production equipment of silicon carbide. Traditional industries, such as the ferroalloy, cement and glass industries, are becoming further compressed or technologically upgraded. On the other hand, we should focus on “enhancing new kinetic energy”, that is, governments at all levels strive to promote the growth of new or advanced industries and reduce the consumption of, and negative impacts on, resources/the environment, such as knowledge-based and technology-based biology, medicine and new material industries. In the past 10 years, industries such as those producing lithium batteries, photovoltaics, biopharmaceuticals and optoelectronic components have developed rapidly in the XMA. Among them, the added value of high-tech industries in Xining accounts for 36.5%, and Xining possess the top ten lithium battery production lines in China. The “one district and four parks” in the Xining Economic and Technological Development Zone are rated as national green parks, and their seven small- and medium-sized enterprises are identified as national-level green enterprises. The total output value of the high-end green building materials in Ledu District Industrial Park reached CNY 678 million in 2018, accounting for approximately 75% of the total output value of the park. High-end equipment manufacturing and new material output accounted for approximately 20%.
(2) Expanding the green industrial chain. The XMA should develop environmentally friendly and profitable industries such as characteristic industries, cultivate a whole green industrial chain and promote its regional integration. This process has strengthened or reshaped the connection between the raw material and processing areas of the industry and changed the spatial organization structure of related industries within the metropolitan area, such as those producing green agricultural and animal husbandry products and their deep processing products, including wolfberry, highland barley and yak with plateau characteristics. This has become a new direction for the green transformation of industries in the XMA.

The “new urbanization” of the XMA is mainly reflected in the following two aspects:

(1) Green-development-oriented population migration: Population migration and urbanization based on the concept of green development. As confirmed by face-to-face interviews, the population urbanization of the XMA has mainly manifested in three major characteristics: the population of Qinghai Province outside the metropolitan area have migrated to the interior of the area, while the population of the inner and outer districts and counties have migrated to the urban area and migrated from the urban areas within the metropolitan area to the main urban area of Xining. People have migrated from mountains and hills to river valleys due to factors such as migrant workers, high altitudes, poor living conditions and poverty alleviation policies [35,41], and the ecological environment of area of origin has been restored through the restoration of farmland to forests and grasslands. For example, the ecological environment of some plateau mountainous and hills is gradually improving due to natural restoration and human intervention restoration, because of the continuous decrease in the population size [42]. Combined with the rapid growth of industries and relevant policies, such as “going out of the city and entering parks”, the XMA has realized industrial spatial agglomeration, and its industrial spatial structure has been reshaped. A typical case of this process is the ecological migration pattern in which people are “forced to migrate because of poverty”. Ecological migration is a systematic state action that functions to eliminate poverty (classes), revitalize the countryside, restore ecology and increase (farmers’) income. The county/township governments in the XMA have relocated to towns or villages or been established in river valleys and plains through the method of “voluntary masses, self-financing by the people, and government investment”. The household registration and the land still belong to the villagers.
(2) Green-oriented integration and improvement of urban and rural development. We must insist on urban system renewal, rural revitalization and the overall planning of urban and rural areas. On the one hand, the government achieves the purpose of systematically improving the living and production environment of residents through the construction of new urban areas/new cities and the renovation of old cities, such as the diversification, quality and high density of the Xining central area. With the joint efforts of the governments at all levels, in 2019, Xining implemented more than 15,000 sets of comprehensive renovation projects for old residential quarters in the cities and towns, while Haidong completed the comprehensive renovation of 2614 urban old residential quarters. On the other hand, the government promotes rural revitalization through the construction of beautiful villages. For example, Huangyuan County adheres to the overall planning and integrated promotion of urban and rural areas and continues to improve the urban and rural appearance and living environment.

### 3.2. “Top-Down” Green Development Drive: A Systematic Management and Control Model with Chinese Characteristics

**Figure 3:**
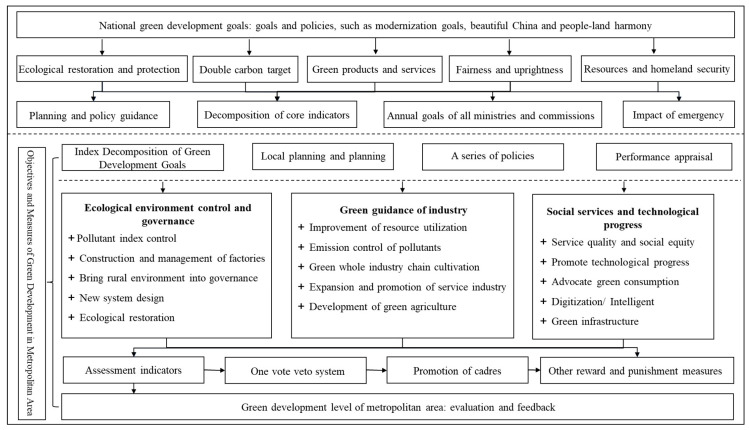
The “Top-Down” Systematic Green Development Management and Control Driving Mode of the XMA.

In underdeveloped areas, the basic driving force for local green development and transformation stems from “upper pressure” regarding national green development goals and policies. For example, based on the plans or policies of the National Development and Reform Commission, the Ministry of Natural Resources, the Ministry of Environmental Protection, the Ministry of Housing and Urban–Rural Development and other ministries, modernization goals, beautiful China and the harmony between humans and the Earth have been “transmitted” to the provincial and municipal levels at an increasing pace through “carbon peaking and carbon neutrality goals”, local green products, (national) ecological barriers, homeland security and land space planning, with the expectation that green development would not only “realize the growth of the metropolitan area under green growth” but also “gradually establish a harmonious human–land relationship in the new era with plateau characteristics”. This has prompted the government and even the metropolitan area to gradually form a grand, comprehensive and government-driven green transformation system model. Based on the green development goals of national and provincial governments, according to the deconstruction of green development indicators, the two cities in the metropolitan area have implemented their own relevant plans, formulated relevant serialization policies and taken into account the impacts of emergencies such as those related to the ecological environment and natural disasters. In reality, the Chinese government has adopted an annual assessment system and included green development goals into this system. Since 2000, green development has gradually become (part of) a key or important performance evaluation or cadre promotion mechanism, especially as a core indicator of cadre promotion and even leadership evaluation. The accompanying reward and punishment measures may affect the future of the “parent officials”. Of course, this institutional design or green institutional shift will drive green actions and their effects in the metropolitan area (Figure 3).

Green development not only involves social and economic transformation and technological progress but also many other issues, such as ecological restoration. It is an immense systematic project. The underdeveloped areas still have problems such as insufficient funds and a lack of related technologies. In response to this problem, we can generally summarize the system-driven model of the XMA as a circular model of “one center, multi-departmental collaboration, grassroots implementation and evaluation and feedback”. Among these components, “one center” refers to the core leadership, especially the spirit of the speeches or guidelines of the main leaders of the central government, provinces and regions, such as the idea of “ecological civilization” construction. “Multi-departmental collaboration” and “grassroots implementation” refer not only to the implementation of the plans by the State Council, provincial and regional departments but also to the implementation, execution and collaboration of the various departments of the city government and grassroots governments at all levels (especially county, district and township governments) in accordance with various plans, programs and indicative spirits. Because green transformation is related to ecological restoration, industrial development, urban construction and other aspects, it is a comprehensive and systematic task. “Evaluation and feedback” refer to the process of identifying problems, providing feedback on problems, evaluating the effects of green development and proposing future “rectification” measures or suggestions according to the annual supervision or inspection results.

**Figure 4:**
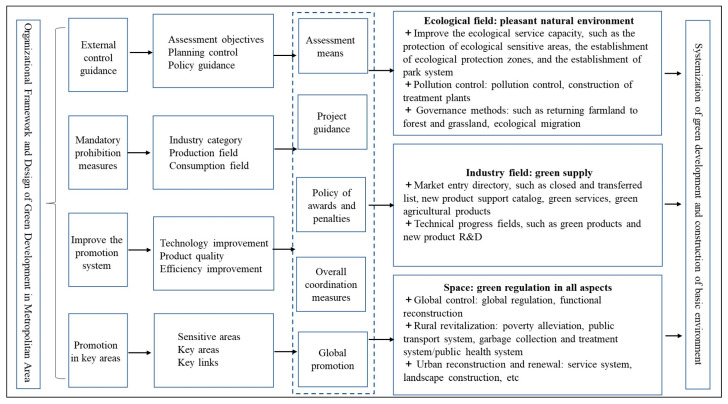
Organizational System and System Design of Green Development Transformation in the XMA.

In general, this green development model is an increasingly systematic, rigorous, oriented, cooperative and global model that is a design and comprehensive system. This system includes an external control subsystem, an internal promotion subsystem, a mandatory prohibition subsystem and a key field promotion subsystem. These systems establish a systematic, three-dimensional green development promotion and improvement system, mainly in the ecological, industrial and space fields, through goal setting, an assessment system, reward and punishment means, global promotion and overall coordination (Figure 4). This can roughly include two systems of external control and internal control: On the one hand, the external control of governments at all levels is mainly limited by explicit and visible goals, mainly including the goals of ecological environment control and governance, such as pollutant index control and ecological restoration areas; industrial green guidance, such as the improvement of resource utilization and pollutant emission control; and social services and technological progress, such as the capacity for technological progress and the green infrastructure ratio. On the other hand, internal control is mainly aimed towards the performance evaluation and promotion of cadres based on annual or tenure assessments, but these are often not disclosed to the public. The key to the success of these measures lies in the green transformation of the industry.

In this way, the management and control driving mode of the XMA can be roughly divided into the following three modules:

(1) Governments at all levels in the metropolitan area have enforced the control and governance of the ecological environment within their jurisdictions, which is also the “hard task” of the central government’s construction of the ecological civilization. This can be summarized as pollutant index control, the construction of treatment factories, integration of the rural environment into the treatment, ecological restoration and innovative system design. Almost all governments at all levels choose to start with the “source” of the pollution, with the following goals: first, to improve the management of binding indicators for energy conservation and emission reduction; second, to create a national water-saving city; third, to improve the economic system of green and low-carbon circular development, accelerate the circular reform of parks, improve the circular economy standards and certification systems, promote green reforms in key areas and key industries and promote circular links between production systems and living systems; and fourth, to build treatment facilities such as pollutant treatment plants, carry out the centralized treatment of pollutants, plan and build an industrial bases for the comprehensive utilization of industrial resources and build a waste resource recovery and recycling system that is integrated online and offline. At the institutional level, the city government has simultaneously performed innovation. Firstly, it has strengthened environmental regulations and the related supervision network, strengthened the prevention and control of pollution from mobile sources and improved the remote sensing monitoring network for motor vehicles. Among them, the environmental regulation of the metropolitan area presents a spatial differentiation of “high center and low edge”. For example, the highest environmental regulation area is concentrated in the main urban area of Xining City, with the greatest pressure regarding environmental improvement, while the peripheral counties and districts are relatively low, which shows that the efficiency of environmental regulation and economic development level creates the spatial coupling [43]. The government’s second aim is to fully implement and improve the “river and lake chief system”, actively promote the comprehensive management of rivers and improve the water quality of nationally controlled fault sections of the river basins. The third aim is to implement environmental protection and treatment projects and promote the joint prevention and control system in Xining and Haidong through, for example, the comprehensive treatment of various emission sources, such as industry, life, agriculture and natural dust. In fact, the various governments of the XMA have strengthened the risk prevention and control, treatment and restoration, and supervision of the development of polluted land and encouraged cross-regional cooperation to build hazardous waste treatment facilities [43,44]. This has yielded good results. For example, the proportion of days with good weather has greatly increased, and heavy pollution has basically been eliminated.
(2) The metropolitan area focuses on the green guidance of the industry. Its core purpose is to improve the utilization rate of resources, cultivate green agriculture and the whole green industrial chain, improve the level of the service industry and reduce the discharge of pollutants according to the control indicators of the ecological environment. In practice, governments at all levels have accelerated the promotion of industrial green development guidance. The first goal is to basically form a circular-economy-oriented green industrial chain with local characteristics; to reduce the use of chemical fertilizers and pesticides and build a low-carbon ecological agriculture and agricultural and livestock product processing industry with the capacity for multi-level recycling (such as Haidong City’s initiative to ensure zero use of chemical fertilizers and pesticides and the active use of farm manure “pure” organic agricultural products in experimental areas); to improve the automation technology level of related industries and the construction of the entire industrial chain; and to integrate production, sales and services so as to expand the industrial chain and extend high-value-added industries (such as the highland barley wine storage business). The second is to create a circular economic system and form an ecological brand of the Qinghai–Tibet Plateau (such as organic fertilizers and plastic film recycling), expand the scale of the plateau’s characteristic industrial chain and drive the green transformation of the plateau’s internal planting industry, tourism development, and resource development in order to drive the formation of a regional spatial division of labor in the entire industrial chain. The third is to establish or develop the local branded industrial chain with the characteristics of the plateau, such as wolfberry and yak as the core, with the green industrial ecological economy characterized by the deep chemical industry of resource-based products, such as characteristic yak and highland barley. The fourth is to rapidly develop the high-tech industrial chain with the core of biology, medicine, lithium battery, etc., and to replace the traditional industries as far as possible.
(3) Metropolitan areas attach importance to social services and technological progress, including a focus on service quality and social equity, the promotion of the introduction of technology and progress, and the promotion of green consumption and comprehensive benefits. The government has paid attention to the integration of local and internal scientific research forces, as well as the introduction, research and development, publicity, trading and brand building of serialized green agricultural products. By cultivating an ecological culture, the city government has increased the supply of green products and services, advocated a green, low-carbon lifestyle and advocated a simple, moderate, green and low-carbon lifestyle. At the same time, the city government has paid attention to the investment and introduction of technology to promote green development, especially in manufacturing, green agriculture and pollution control. For example, the city government has attempted to develop green agricultural products and organic agriculture by establishing agricultural industrial parks and other means. The green development of agriculture takes circularization, organization, specialization, chain extension and internalization as the basic paths to promote increases in farmers’ incomes, rural poverty alleviation and urban–rural integration. The city government “forcibly promotes” the production mode of agricultural products without chemical fertilizers, replacing chemical fertilizers with organic fertilizers, reducing agricultural pollution and actively building a green brand of organic agricultural products. On the other hand, agricultural development has also strengthened the integration of production–processing–sales and deep integration with the cultural tourism industry. For example, highland barley wine and other wine industries extend the industrial chain from agricultural product processing to form high-value-added industries, such as the Tianyoude Highland Barley Distillery, by attaching importance to cultural exhibitions and integration to form the unique culture of products and enterprises, establishing cultural exhibition halls in enterprises, and carrying out the wine storage businesses so that they complement each other through product sales. Thus, some enterprises have built new corporate spaces and formed their brand values and unique cultures. In addition, a few enterprises have set up highland barley and other planting bases outside the metropolitan area to regionalize the production chain. Therefore, the enterprises increase the added value of their products by paying attention to the combination of agriculture, animal husbandry, e-commerce and Internet+ and, especially, integrating e-commerce into agricultural development. The processing of branded agricultural products can be complemented with online promotion and online/offline sales. The enterprise can introduce the Industrial Internet into the slaughtering industry and combine it with the Internet APP to create an internet information platform for the slaughtering industry.

## 4. Actors and Basic Characteristics of the Green Development Transformation in the XMA

Green development is a systematic and comprehensive task. After our investigation, we came to realize that the direct driver of the green development of the XMA is the government at all levels, while enterprises and residents/village are willing or unwilling passive performers, and the media are based on the government. A booster of “green development public opinion” is required by enterprises and society. Usually, cooperation between governments, enterprises and the public is an important means through which actors can jointly promote local green development.

### 4.1. All levels of Government: Core Leaders, Facilitators and Monitors

First, governments at all levels are the core actors of green development. On the whole, green guidance based on interest trends is easy to implement, while mandatory policy implementation resistance is relatively large. However, only the government can assume the “mandatory role” of this advanced green development. Taking advantage of the socialist system with Chinese characteristics, the XMA has adopted the method of “subcontracting at different levels” within the administrative area to promote green development. For example, it has carried out administrative-stratified target assessments and even a “river chief system” for the problem of river pollution.

Green development often requires mandatory methods in the public domain, such as mandatory ecological protection and governance, the adjustment of the structure of currently consumed products, reasonable investment and distribution, etc. Many officials clearly acknowledge that the implementation of green development policies or projects based on public interests often requires coercive means, mainly including ecological protection and restoration, companies’ efforts to reduce pollutants and foster technological improvements due to the need for “emission reduction”, the prohibition of the mining of similar minerals or river sand for building materials and the prohibition of the production or sale of certain industries or products, etc. Although the implementation of these policies is beneficial for public interests such as ecological protection, “it often damages the vital interests of relevant enterprises, people or localities. If there is no coercion, relying only on appeals will have almost no substantive effect”. A middle-aged male interviewee said, “The central government pushed the concept of green development in China so that it became a wealthy society like the West. However, in fact, (Qinghai Province) is in the interior of China, and the level of social and economic development is in a backward state and requires a large amount of transfer payments from the state every year to ensure the payment of wages and infrastructure”. At the same time, the leaders/supervisors of most of the cities, counties and districts in the XMA almost believed that relying solely on local economic strength and finance cannot promote or complete the green transformation, because the efficiency and profit of the local economy are generally low, and the finance cannot be self-sufficient, while green development is a “higher” development requirement and requires corresponding economic support and technical capabilities. A male interview official said, “Even with the support of a few enterprises and counties and towns, green development cannot be achieved”. Moreover, since the Reform and Opening Up, cadres at all levels have been “accustomed” to the traditional development model that relies on high investment and high consumption. The mentality of enterprises and the public as being “indifferent” to the ecological environment is relatively strong, and they cannot force entrepreneurs and the public to exhibit “voluntary” commitment and green shifts.

The XMA has gradually promoted the ecological restoration and environmental governance of the whole area on the urban scale and carried out the relatively systematic governance of key problems and areas [43,44]. A number of large or important environmental protection and ecological restoration projects have efficiently been completed, including: ① the “Three North” shelterbelt construction, that is, the million-mu artificial forest base in the Huangshui River Basin, through the greening of the North and South mountains and restoration of some farmlands to forests and grasslands; the “four waters at the same time”, that is, “the governance or maintenance of water resources, water ecology, water environments, water disasters” by carrying out special treatments for prominent problems such as soil and water conservation and illegal sand mining along the Yellow River. Thus, the deterioration of the ecological environment has been curbed, and the effect of increasing greenery and the residents’ incomes in the region is obvious. On the city scale, Xining City has carried out greening actions for beautiful homes, such as the construction of a greening network of highways through the city known as the North–South Line of the Airport Expressway and implementation of projects such as “Tianbao”, “Public Forests” and “the North and South Mountains Green”, planting 1.11 million acres. The forest coverage rate is up to 32%. However, most of these tasks are completed through the input of the state/local government and mandatory policy measures. Correspondingly, the government mainly adopts induction methods to promote green development in non-public areas. If the source of funds is reliable or secure, the government will be more willing to use inducement or guidance to promote green development. These methods generally include the following: firstly, providing all or part of the funds for technological transformation, ecological restoration, to induce or “force” enterprises or grassroot units to provide part of the funds and labor, and to achieve green development goals through technological transformation projects; secondly, the approval of relevant projects and attachment of green development conditions, such as industrial access, new product production and new district construction, with the approved construction of these projects adding new relevant provisions for green development; thirdly, setting up special green development projects to directly promote and drive a certain aspect of the green shift, such as the establishment of demonstration wetland parks, standardized farmland, etc., in order to drive and demonstrate how to carry out green development; and fourthly, creating a social atmosphere and values of green development and forming a general social trend of green development.

**Figure 5:**
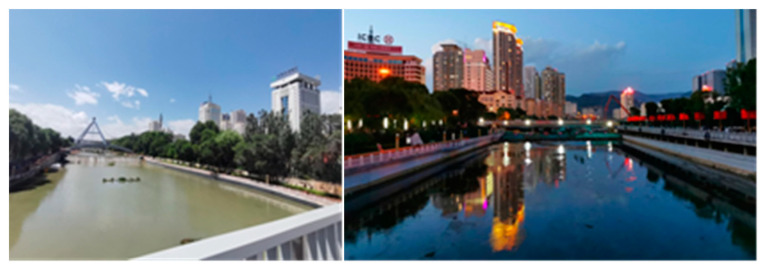
Current Riverside Street View of Xining.

**Figure 6:**
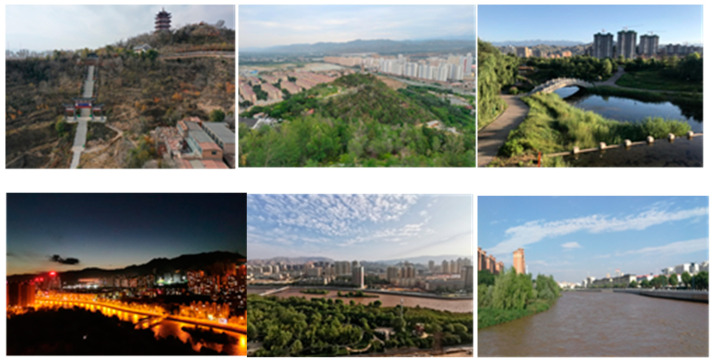
Mountain Greening and Modern Urban Form in Ledu District, Haidong.

Having “Leaders focus, keep paying attention, and focus on solving” problems is the key to the green development of the XMA. On the one hand, because green transformation is a certain degree of “advanced” behavior, documents and plans alone may not achieve the expected results. On the other hand, based on its limited funds, the XMA implements key projects to solve difficult problems or so-called pain points, for example, by gradually strengthening various special governance and improvement projects, that is, overcoming the main contradictions or key problems involved in green development. For example, pollution control compliance, service industry upgrading, new industry development, green product research and development, inspection and testing, ecological restoration, etc., are all areas or major events that core leaders must focus on. The most typical one is ecological restoration and governance, promoting the construction of ecological barriers in the arid mountainous areas of eastern Qinghai. Driven by the attention and continuous investment of previous governments, the XMA has attached great importance to ecological restoration, implemented global ecological governance, built green plateau areas and shaped the “Plateau Landscape Metropolis Circle”. That is, the city government has launched and implemented action plans such as “Plateau Green” and “Green City” to build an ecological security pattern. For example, a demonstration area, namely the Sanjiangyuan characteristic cultural heritage area known as the Qinghai–Tibet Plateau wildlife science area, has emphasized the creation of a natural reserve city model of harmonious coexistence between humankind and nature. The grassland area in the XMA increased from 11,554.71 km^2^ to 11,561.45 km^2^ from 2000 to 2020 [34]. Xining is the only provincial capital city in Northwest China that has won the titles of “National Garden City” and “National Forest City”. The urban forest coverage rate is 35.1%, the green coverage rate in the built-up area is 40.5%, and the park green space area per capita is 12.5 m^2^. The urban mountain greening of Xining City is a typical case of “successive governments, constant efforts”. The greening project of the two mountains in the north and south is a landmark demonstration project of ecological civilization construction in Qinghai Province. The greening area is large. The Nanshan starts at Yanggou Bay in the east and ends at Xishan Bay in the west. The Beishan starts at Yunjiakou in the east and ends with the Xiaoyou Mountain in the west. The forest coverage rate ranges from 7.2% to 79%, and the barren hills and ridges that used to be “blown by the wind and the sand fly without the shadow of birds” have become lush, with birds and the fragrance of flowers (Figure 5). The two mountains in the north and south of Haidong City start at Xiaoxia at the junction of Ping’an District and Xining City in the west and extend to Minhe County and Honggu District of Lanzhou City, Gansu Province, in the east, with a length of approximately 107 km from east to west. Starting at the foot of the mountain and progressing toward the first-class ridge line, the average width is approximately 1.6 km. Since the project was launched in 2013, a total of CNY 302 million has been invested, the afforestation area has reached 1527 hectares, the greening area has reached 20 km^2^, and more than 15 million high-specification seedlings have been planted. It plays an irreplaceable role in improving the living environment, expanding the living space and developing leisure, sightseeing and tourism. Among the various areas, Chaoyangshan Park in Ledu District has also carried out “soil replacement” work because of its thin or poor soil, and the vegetation has grown (Figure 6).

### 4.2. Various Enterprises: Key Performers and Responders

Enterprises are the main executors of the green development of metropolitan areas, and the greening of enterprises is the result of the transformation of green development. From the perspective of changes in corporate profits, the speed of the overall corporate profits and industrial upgrading is the basic criterion for testing whether or not the green development of metropolitan areas can be sustainable.

Generally speaking, the enterprises in the XMA can be divided into the following categories according to their green development: Firstly, traditional industries, such as extraction industries and iron and steel industries, are often crude, large, and large-scale polluting enterprises based on the poor use of resources. These enterprises have low profits and cannot afford the operating costs of environmental pollution control (even if they have control equipment, they cannot operate normally). Most of the interviewed enterprise managers and officials of the Environmental Protection Agency have admitted that these enterprises could not undertake green transformation by themselves. However, if the government shares the capital or technology for green transformation with them, these enterprises could not oppose green development policies. Secondly, high-tech industries, characteristic industries, service industries, agricultural enterprises or industries with relatively high environmental requirements and relatively high profits are willing to devote themselves to green development but also require financial assistance from the government to varying degrees (in fact, the development environment of the development zone has provided the green development conditions for these enterprises, such as the circular economy and infrastructure). Among the categories of China’s “Strategic Emerging Industries Classification (2012)” (trial), there are six types of strategic emerging industries in the XMA that are growing. For example, the energy conservation and environmental protection industry was established from scratch in 2018. The output value of the new generation information technology industry is approximately CNY 5 billion, the output value of the biological industry exceeds CNY 1 billion, the output value of the high-end equipment manufacturing industry exceeds CNY 10 billion, the output value of the new energy industry exceeds CNY 12 billion, and the output value of the new material industry is approximately CNY 55 billion, which will benefit the industry. In addition, according to the 2021 “Xining Municipal Government Work Report”, during the “13th Five-Year Plan” period in Xining City, the development of strategic emerging industries will accelerate, and the added value of the above-scale industries will increase by 7.4% annually, while the average annual added value of new energy and high-tech industries will increase. They have increased by 54.4% and 19.8%, respectively, and the proportion of strategic emerging industries among the above-designated industries has increased from 5.1% to 24.7%. The third point to note is that for companies between the above two categories, if there are no government mandatory requirements or sources of funding, there will be relatively few companies that can voluntarily undertake green transformation. However, new enterprises “patented” by green transformation policies or old enterprises that have improved their own benefits because of green development often support the government’s green policies, such as enterprises producing green building materials. In general, the attitudes of enterprises are relatively differentiated because they involve the fundamental interests and even survival conditions of enterprises. In fact, a rising number of companies are increasingly affected or pressured by green development policies and are actively taking countermeasures, including the adoption of new technologies, to reduce pollution and developing new green products.

### 4.3. Citizens and Media: Decentralized Doers and Receivers

Numerous respondents admitted or tacitly agreed that residents/townships have a very clear attitude towards green development. That is, as long as the economic income of individuals or families is not reduced and more comprehensive “benefits” are provided, they are all willing to support the government’s green development. Development policies such as the creation of beautiful fields, a suitable environment and safe food are options that are strongly supported by the public. On the other hand, if the green development increases the additional expenses of households and exceeds the range of “sustainable” or “support”, the attitude of these people will be relatively vague or they will ne inclined to let the government bear these “extra expenses”.

For this social class, “persuasion and education” and “interest induction” are common means of green development. Therefore, the government pays attention to the people’s livelihood and pays attention to the sharing of benefits, which is a feasible method for green development. By paying attention to the people’s livelihood, governments at all levels can solve the core issues that the people are concerned about and obtain the support of the people for green development concepts and actions, such as improving transportation, sanitation and other conditions, disposing domestic waste for free and solving poverty problems such as the “Blue Sky Project” (with air pollution prevention and control actions focusing on dust suppression, coal reduction, gas control, vehicle control and combustion bans), “clear water project” (urban and rural sewage treatment, the full coverage of the urban sewage pipe network and promotion of centralized and decentralized sewage treatment modes) and “purifying soil engineering” (soil pollution prevention and control, agricultural land classification management, construction land access management, the implementation of the “plastic restriction order”, solid waste reduction and recycling and safe engineering, as well as the treatment of salt water in the Yellow River area of alkalized land, Yuanshi Mountain polluted land restoration, mine greening restoration and comprehensive treatment of tailing land), so that “the masses can feel the benefits of green development, support green development policies, and consciously change their own concepts”. In fact, most of the people interviewed supported projects that are provided free of charge, such as garbage disposal, rural public transport systems and the supply of clean energy. In addition, the government pays great attention to the construction of infrastructure and invests a great deal of funds, and the public appreciates such “convenience projects”. By the end of 2019, all 354 administrative villages of the 19 townships (towns) in Ledu District had smooth traffic, the road unobstructed rate was 100%, and the village road hardening rate reached 100%. Currently, there are 29 existing passenger lines covering 19 towns and 168 villages in the district. There are 15 bus lines covering 12 towns and 186 administrative villages, and the bus coverage rate of the administrative villages is 52.5%.

Usually, the government influences public opinion through newspapers, news, self-media, etc., publicizes policies and enhances legal awareness of green development so that the public can see and realize that “green mountains and clear waters are invaluable assets”. In fact, the ever-improving ecological environment has also led people to believe that green development is indeed necessary and that it is also conducive to everyone’s health and happiness. For example, people admit that “(Now) the water is clearer, the mountains are greener, and life is better, the government did the right thing”.

## 5. Discussion

China’s economically developed regions are realizing a win-win situation of fostering the ecological environment and economic development on the road toward green development. For example, Zhejiang Province is focusing on eco-tourism to develop the green economy. As early as 2002, Fujian Province proposed the goal of establishing an ecological province. Green communities and green schools are everywhere. Shanghai vigorously implements policies and measures such as the “green office” and “green organ” policies and starts with the government itself in promoting the overall green development of the region. Hainan Province strives to build an international eco-tourism island. However, Western China is restricted by the concept of realizing green development, capital, technology, talent barriers and institutional restrictions, and its green development path is fundamentally different from that of economically developed regions. In view of these differences, the XMA proposed the “Western Plan”.

### 5.1. The Green Development Level of the XMA

Based on the guidance and drive of the government, the XMA has established the green development goal of “green, efficient and promotion” and is accelerating the industrial restructuring and establishment of a characteristic industrial system, strengthening the cooperation between counties and districts in the region and ecological restoration and forming a localized green development strategy. Based on six first-level indicators of resource utilization, environmental governance, environmental quality, ecological protection, growth quality and green life, the green development level of the XMA in the past 20 years was assessed [35]. Firstly, on the whole, the green development level of the XMA has been greatly improved, but it is still at a relatively early development stage. The spatial pattern is generally characterized by a high level in the northeast and a low level in the southwest, that is, the level of green development tends to be significantly polarized between high-value areas and low-value areas, which reflects the circular cumulative effect of green development space in the metropolitan area. Secondly, the environmental quality index is generally high and shows a continuously increasing trend. This is because the government has continuously improved its environmental regulation and implemented ecological protection and restoration projects, ecological demonstration projects, the joint prevention and control of pollution and other environmental protection and governance projects.

**Figure 7:**
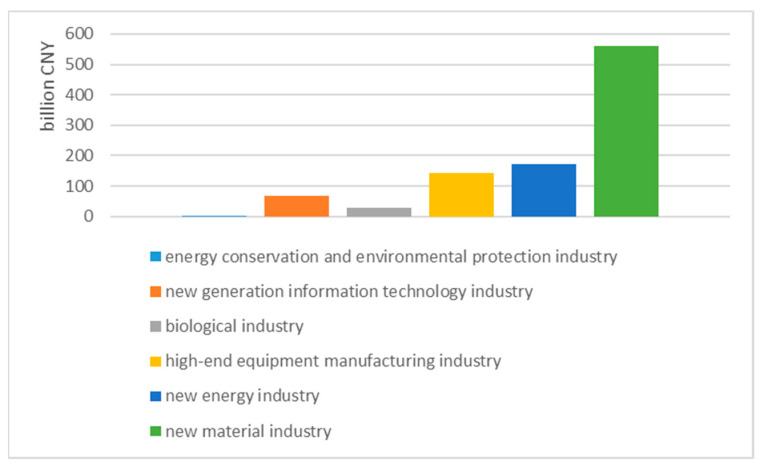
Strategic New Industrial Output Value of the XMA in 2018.

A notable feature is that the output value structure of the three industries in the XMA has undergone historic changes from 2000 to 2020. The proportion of the output value of the primary industry has shown a slow downward trend, and since 2017, the three industrial structures in the XMA have generally shown a structural trend of “three, two and one”. Moreover, according to the Classification of Strategic Emerging Industries (2012) (for trial implementation), the XMA has actively developed six categories of strategic emerging industries: the energy conservation and environmental protection industry, the new-generation information technology industry, biological industry, high-end equipment manufacturing industry, new energy industry and the new materials industry. Among them, the strategic new industries, service industries and financial industries have gradually become the leading industries in the XMA (Figure 7).

### 5.2. The Socialist System with Chinese Characteristics Is the Institutional Guarantee for the Transformation of Green Development in Underdeveloped Areas

According to the survey, the XMA has made great achievements in green development, but the endogenous driving force for green development is still poor or even lacking. Only the advantages of the socialist system with Chinese characteristics can promote or guarantee this advanced green transformation. In reality, the green development of the XMA has been fully supported by the state, including state transfer payments and “correspondence support” for the construction of major infrastructure services (roads, railways, communications and water conservancy), ecological restoration and protection, and education and technical support. The “free” continuous investment of these countries is the fundamental condition for the transformation of green development in this area. Therefore, the essence of green development is the process of replacing the traditional economic system with a new economic system, which requires relatively strong financial resources in order to guarantee it. That is, the green transition of the XMA is a unique process, and it is also possible that it could “stagnate” or “return to a non-green state”. Although the XMA belongs to the “first-mover area” in Qinghai Province, it is in the stage of rapid growth, but it is still in the middle/late stage of industrialization. The industrial upgrading must be completed urgently, and it cannot spontaneously transform to a green development state to a certain extent. At present, the industrial structure system of the XMA is still relatively backward. For example, the pillar industries are still dominated by traditional metal smelting and heavy chemical enterprises, which have resource advantages but have not yet formed a characteristic manufacturing and intelligent industrial chain. Civilization construction requires relatively difficult steps. The high-tech industries and green industries are too small in scale, and the industries are local and seasonal. For example, the raw materials for manufacturing are mostly plateau animals and plants and other unique resources, and the tourism services are mainly based on the ethnic culture and plateau landscapes. The substitution effect of green development cannot be produced. In reality, many officials admitted that because of “the weak economic foundation, low profits, and weak support for the industry as a whole for green development), the XMA is totally lacking intrinsic (green development) motivation”.

### 5.3. “Top-Down” Promotion and Differences between Actors

Strictly speaking, the green development of the XMA is not driven by market power (although it is undeniable that the market power is extremely slow but increasing) but by a top-down “seeing hand”. As a result, the green development of the XMA cannot be “comprehensive” but focuses more on “visible and tangible” factors such as production, energy, ecology, the environment and spatial organization, which can reflect “government achievements”. In the macro-field, at least in the short term, it is unlikely to penetrate the deep field. For example, green building materials cannot be sold in large quantities without government support because of their relatively high prices. In fact, the areas undergoing fast green development are often the areas that the government pays attention to and invest more in, while private forces tend to focus on green products or areas that can create more “profits”, such as dairy products, wine and fruits.

In reality, there are significant differences between the actors involved in the green development of metropolitan areas. Firstly, the central government is the active leader, while the provincial and municipal governments are the promoters and executors, and most county/township governments are also the executors. Because governments at all levels have different economic capabilities, especially financial capabilities, their attitudes towards green development are different. Generally, projects or plans that receive various support from higher-level governments, have strong financial capabilities and can provide certain benefits through green development are welcomed by grassroots governments and vice versa. Clearly, not all township governments are willing to promote green development “regardless of cost”. Secondly, the actor of the enterprise is more dependent on the orientation of interests. Green development can increase or at least not reduce the profits of enterprises, and it will definitely be supported by enterprises. Even if it is a mandatory policy or system, a company may implement it in a “flawless way”, and the lowest yield is its bottom line. Otherwise, it may lead to bankruptcy. Of course, the industries or enterprises listed in the “shut down and transfer” list are enforced and must be “victims” of green development. In the short term, this will obviously affect local GDP, employment and fiscal revenue, etc. Thirdly, at the social level, the vast majority of people are, of course, willing to enjoy the benefits of green development, such as beautiful environments, parks, clean rivers and villages, but they are less willing to take on more responsibilities and costs. Therefore, the public’s concept of green construction and consumption has not been fundamentally established. For example, they are most sensitive to product prices, and the public (with the exception of high-income earners) and the market currently do not pay special attention to whether or not a product is green.

Therefore, this government-driven model means that in less developed regions, the government has become the core driver of green development, at least in the current stage of development. This has led all sectors of society to take it for granted or habitually believe that green development is mainly the responsibility and obligation of the government. During our field research, we found that the government planned, promoted and implemented the design and actions of green development, while the enterprises only partially assumed their part either passively or when driven by interests, and the people were generally the beneficiaries.

### 5.4. Systematic Green Development Still Requires Continuous External Support and Intra-Domain Collaboration

According to the survey and interviews, government officials at all levels generally admit that with the “deepening” (such as green industry development and ecological governance) and “generalization” (whereby the field of green development becomes increasingly extensive) of green development, “the more funds that are needed, the longer it takes, and the more difficult the transition will be“. Clearly, the local level of economic strength is not sufficient to support this green transformation process, and there is no doubt that continuous support from the central government and other funds, technologies, and personnel is required. This external intervention is clearly the key driving factor for the green transformation of underdeveloped areas, including the goals, funds, technology and planning guidance.

A key issue worthy of attention is that almost all projects are designed and implemented within urban administrative districts, and at least on the watershed scale, there are discordant phenomena such as land use, ecological restoration and pollution control. Therefore, the XMA still needs to carry out intra-regional coordination at two levels [35,45]. The first is the systematic coordination of green actions. The metropolitan area should continue to strengthen the institutional, financial and technical guarantees for green development, promote the green transformation of key industries and fields and integrate the ecological and environmental projects in Xining and Haidong at the metropolitan level, such as greening projects, environmental governance and rural revitalization. The second is coordinated spatial planning and development based on green development goals on the metropolitan scale.

According to the “14th Five-Year Plan” of Xining, Haidong and their districts and counties, the metropolitan area should at least coordinate the secondary industry as follows. The first focus is the construction of new industrial development bases, such as the cultivation of high-altitude biomedicine, healthcare, big data and intelligent manufacturing industrial clusters and construction of Xining Polar Innovation City, which has become a source of high-quality development in the province. Haidong Industrial Park has been upgraded to a national economic and technological development zone, focusing on the construction of high-end equipment, electronic information, new materials and new energy. The second is the upgrading of traditional industries, such as the traditional industry parks in Xining (such as the Ganhe Industry in Xining) and Haidong (such as the metal smelting and building materials in the park), and their transformation into high-end, intelligent and green industries. Regarding the specialization and greening of agriculture, the XMA should also adapt to local conditions and promote regional synergy.

## 6. Conclusions

Thus far, capitalist countries have achieved the goal of green development by means of increasingly stringent environmental protection regulations, technological upgrading, industrial upgrading and global transfer based on market mechanisms and legal environments. However, since 2000, the XMA, located in the underdeveloped western part of China, a large developing country, has gradually formed a new model of green development in underdeveloped areas that has been strongly driven by the government since 2000. Under the influence of this mode, the XMA has gradually promoted the ecological restoration and environmental governance of the whole area on the urban scale over recent years, and a number of large or important environmental protection and ecological restoration projects have efficiently been completed. An increasing number of companies are actively taking counter measures in order to respond to green development policies. The vigorous publicity of government policies and the constantly improving ecological environment have also greatly enhanced the public’s legal awareness of green development. This mode is mainly reflected in the following ways:

Firstly, this is a new model of green development that integrates internal and external factors, that is, a green development path based on the local development stage of production and life, industrialization, urbanization, human activities and ecological environmental protection that promote each other and cyclically upgrade. During the period of rapid growth, the areas of the XMA have promoted each other through new urbanization and new industrialization and jointly promoted the formation of a green development turn in the new era.

Secondly, green development is a systematic and comprehensive task and an immense systematic project, which can be summarized as a circular model of “one center, multi-departmental collaboration, grass-roots implementation, evaluation and feedback”. This model is a systematic, cooperative and global system design and comprehensive system, which is mainly reflected in the ecological field, industrial field and space field. This roughly includes two systems of external control and internal control: one is the external control of governments at all levels, which is mainly limited by explicit and visible goals, primarily including the goals of ecological environment control and governance, while the other is internal control, which is mainly annual or lasting for one term of office. The performance evaluation and promotion measures of the cadres in the assessment are often not disclosed to the public. Because it is in an underdeveloped state, in order to achieve the path of “development concurrent with governance”, the formulation and implementation of green policies are often forced, mainly being reflected in aspects such as industry, ecology, the environment, space and transportation.

Thirdly, the direct drivers of green development are governments at all levels, while enterprises and residents/village are mostly passive executors, whether voluntarily or involuntarily, and the media tend more to shape “public opinion on green development” based on the needs of the government, enterprises and society. Governments at all levels are the core actors of green development. That is, only the government can assume the “mandatory role” of green development that is ahead of its time. Enterprises rely more on profit orientation and even coercive means. This class of people are more willing to enjoy the benefits provided by green development and tend to let the government bear the “extra expenditure” of green development. Therefore, persuasion, education and the induction of interest are reliable means with which to persuade them to switch to green development. Therefore, the government pays attention to people’s livelihood and pays attention to the sharing of benefits, which is a feasible method of green development. Therefore, collaboration between governments, businesses and citizens is very important for the green transition.

Fourthly, the advantage of the socialist system with Chinese characteristics is the guarantee of the green development model. Mainland China is a political and economic system with reasonable decentralization of its central and local governments. The unique institutional advantages enable the green shift to the sustainable development strategy of the central government, which is implemented by the State Council and provincial, municipal and district governments. In reality, the Chinese government has adopted an annual assessment system and has included green development goals in this assessment system, such as key performance considerations or cadre promotion mechanisms. Therefore, on the premise that the endogenous power of green development is still lacking, the institutional advantages of socialism with Chinese characteristics guarantee this advanced green transformation process. In addition, local green development has also received payments transferred from the national finance and the “counterpart support” from the provinces and regions, including the support or assistance of major infrastructure service facilities, ecological restoration and protection, education and technology.

It is worth noting that the green transformation requires a great deal of “additional” investment, industrial upgrading and ecological governance. It also requires time and the understanding and assistance of individuals across all sectors of life. Therefore, future research requires more in-depth analysis of cost–benefit changes and the cognitive changes among all social classes. Of course, the inherent contradictions of green transformation still need to be further revealed and analyzed.

## Data Availability

Not applicable.
